# Is pre-Darwinian evolution plausible?

**DOI:** 10.1186/s13062-018-0216-7

**Published:** 2018-09-21

**Authors:** Marc Tessera

**Affiliations:** Meudon, France

**Keywords:** Metabolism-first, Replicator-first, Combined metabolism-replicator scenarios, Pre-Darwinian evolution, Prebiotic evolution, Darwinian evolution, Origin of life, Origin of Darwinian evolution

## Abstract

**Background:**

This essay highlights critical aspects of the plausibility of pre-Darwinian evolution. It is based on a critical review of some better-known open, far-from-equilibrium system-based scenarios supposed to explain processes that took place before Darwinian evolution had emerged and that resulted in the origin of the first systems capable of Darwinian evolution. The researchers’ responses to eight crucial questions are reviewed. The majority of the researchers claim that there would have been an evolutionary continuity between chemistry and “*biology*”. A key question is how did this evolution begin before Darwinian evolution had begun? In other words the question is whether pre-Darwinian evolution is plausible.

**Results:**

Strengths and weaknesses of the reviewed scenarios are presented. They are distinguished between metabolism-first, replicator-first and combined metabolism-replicator models. The metabolism-first scenarios show major issues, the worst concerns heredity and chirality. Although the replicator-first scenarios answer the heredity question they have their own problems, notably chirality. Among the reviewed combined metabolism-replicator models, one shows the fewest issues. In particular, it seems to answer the chiral question, and eventually implies Darwinian evolution from the very beginning. Its main hypothesis needs to be validated with experimental data.

**Conclusion:**

From this critical review it is that the concept of “*pre-Darwinian evolution*” appears questionable, in particular because it is unlikely if not impossible that any evolution in complexity over time may work without multiplication and heritability allowing the emergence of genetically and ecologically diverse lineages on which natural selection may operate. Only Darwinian evolution could have led to such an evolution. Thus, Pre-Darwinian evolution is not plausible according to the author. Surely, the answer to the question posed in the title is a prerequisite to the understanding of the origin of Darwinian evolution.

**Reviewers:**

This article was reviewed by Purificacion Lopez-Garcia, Anthony Poole, Doron Lancet, and Thomas Dandekar.

## Background

This essay highlights critical aspects of the plausibility of pre-Darwinian evolution. It is based on a critical review of some of the better-known open far-from-equilibrium system-based scenarios supposed to explain the likely processes that took place in times when Darwinian evolution had not yet emerged and resulted in the origin of the first systems capable of Darwinian evolution. Among the current views about the origin of “life” a phase of “prebiotic” chemical evolution is contemplated before the emergence of biological evolution, which the author prefers to call Darwinian evolution. According to those researchers that propose “*prebiotic evolution*”, this describes the chemical processes that took place on the “*prebiotic Earth*” about 4500 to 3500 mya ago. These events preceded biological evolution, a phase which led to the appearance of first “*living cells*” capable of self-reproduction at the expense of some rudimentary metabolism [1]. According to this view the study of the “*origin of life*”’ is an effort to understand the transition from chemistry to biology, a fundamental transition and a lengthy pathway comprising many stages, each of which is the subject of numerous scientific questions [2].

This would imply an evolutionary continuity between chemistry and biology [3–6]. The question then becomes how such a disordered (high entropy) primordial cell system can be re-organized into an evolving “*living state*”, or whether there are compelling theoretical or technical reasons why this is impossible [3].

In other words how did evolution begin if the complex machinery for Darwinian evolution was not in place? [2]. When the emergence of Darwinian evolution is considered (often cited as a key aspect of the definition of life with good reason, as Darwinian evolution is indeed the unifying characteristic of all of biology) once cells with genetically encoded advantageous functions existed, classically defined Darwinian evolution had begun according to J. Szostak. But what is about the previous steps? [7]. Actually the expression “*pre-Darwinian evolution*” will be preferred in this article instead of “*chemical evolution*” or “*prebiotic evolution*” except when the researchers the author cites used them. According to the author Darwinian evolution is a mechanism that can be clearly defined while the concept of life is questionable [8–12]. As the concept of pre-Darwinian evolution is debatable (and questioned in this paper), such an evolution is only defined in reference to Darwinian evolution, i.e., as a chemical or metabolic evolution in complexity over time before Darwinian evolution had emerged. Systems with the ability of Darwinian evolution should have the following characteristics according to the author (1) be open far-from-equilibrium systems able to remain far-from-equilibrium by feeding from their environment; (2) capable of multiplication into similar systems, and (3) of heritability [10].

## Pre-Darwinian or Darwinian evolution scenarios?

Most authors support the idea that only open far-from-equilibrium systems are able to show “*chemical evolution*”, pre-Darwinian evolution according to this author’s usage. Here, only seeming plausible scenarios or models within the paradigm of open far-from-equilibrium systems are reviewed. Each model is evaluated according to the following questions: (1) What was the initial chemical substrate? (2) Are there experimental data supporting the main hypothesis? (3) What was the energy source? (4) Is the chiral question solved? (5) What is the ability of multiplication of the systems? (6) Is there any heredity? (7) Were there plausible site(s) where the initial chemical substrates might arise? (8) Is the evolutionary path plausible? (Table 1: Strengths and weaknesses of the reviewed scenarios for the origin of Darwinian evolution). The majority of the researchers highlight the chiral question. For example József Garay claims that any theory of origin of life that does not explain the origin of homochirality in biomolecules is not complete [13].

**Table 1:** Strengths and weaknesses of the reviewed scenarios for the origin of Darwinian evolution

Scenarios		Initial Chemical Substrate	Experimental Data on main Hypothesis	Energy Source	Chiral Question	Multiplication abilities	Heredity	Plausible Sites	Evolutionary Path	Initial Evolution Claimed
Metabolism-First	Iron-sulfur world	Pioneer organism	Lacking	Volcanic-hydrothermal fluids	Not an issue according to the author	Not truly present	Not clearly demonstrated	Volcanic aquifers	Questionable	Pre-Darwinian
	Vasas et al.	Lacking	Lacking	Not specified	Not addressed	Present	Abstract	Not specified	Plausible	Darwinian
	Fernando & Rowe	Lipid vesicles	Lacking	Solar	Not addressed	Present	Some	Not specified	Complex	Pre-Darwinian
	Russell	Inorganic membranes	Preliminary	White smokers	Not addressed	Unknown	No	White smokers	Questionable	Pre-Darwinian
Replicator-First	RNA/Pre-RNA world	RNA or pre-RNA	Present	Not specified	Not addressed	Present	Present	Not specified	Plausible	Pre-Darwinian
Combined	DKS	Lacking	Lacking	Solar	Not addressed	Not truly present	Not addressed	Not clearly specified	Questionable	Darwinian
	Lancet & Segré	Lipid micelles	Lacking	Volcanoes, hydrothermal vents, solar	Not an issue according to the author	Present	Questionable	Volcanoes, hydrothermal vents, solar	Questionable	Pre-Darwinian
	Tessera	Lipid vesicles	Lacking	White smokers	Possibly solved	Present	Present	White smokers	Possibly handicapped in the polymerization process	Darwinian

It is important to distinguish between metabolism-first, replicator-first and combined metabolism-replicator models. The metabolism-first hypothesis means that ordered chemical reactions, and not replication was the property of the initial form that led to “*life*” and the interlocked networks of chemical reactions that evolved in complexity over time. Replicator-first scenarios require both replication and at least some heredity. Finally it will be attempted to specify which initial evolution is hypothesized by the authors in their model, i.e., pre-Darwinian or Darwinian.

## Metabolism-first scenarios

### Iron-sulfur world model

Wächtershäuser proposes what he calls the “*FeS World theory*”, also called the “*iron-sulfur world*.” This posits that life began as an autotrophic metabolism in hot volcanic-hydrothermal fluids and evolved with organic products turning into ligands for transition metal catalysts thereby eliciting feedback and feed-forward effects. It postulates a “*pioneer organism*” at sites of reducing volcanic exhalations. The pioneer organism is characterized by a composite structure with an inorganic substructure and an organic superstructure. Within the surfaces of the inorganic substructure iron, cobalt, nickel and other transition metal centres with sulphido, carbonyl and other ligands were catalytically active and promoted the growth of the organic superstructure through carbon fixation, driven by the reducing potential of the volcanic exhalations. This pioneer metabolism would reproduce by an autocatalytic feedback mechanism. Some synthetic organic products would have exhibited autocatalytic positive feedback into the synthetic reactions that produced them, which equates to reproduction according to this author. Other organic products served as ligands for activating catalytic metal centres from whence they arose. In this context Wächtershäuser posits that the three polymers of the genetic machinery essentially coevolved from monomers through oligomers to polymers. According to Wächtershäuser, the unitary structure–function relationship of the pioneer organism later gave rise to two major strands of evolution: cellularization and the emergence of the genetic machinery [14]. Regarding chirality Wächtershäuser makes several postulates: (i) the enzymes for the synthesis of the chiral phosphoglycerol lipids of the pre-cells were not enantio-specific and therefore generated racemic lipids; (ii) the racemic lipid membranes of the pre-cells continuously underwent spontaneous symmetry breaking by spatial lipid segregation into an interdigitated micro-pattern of two homochiral lipid domains within each pre-cell membrane; (iii) the racemic pre-cell membrane, while not as stable as a homochiral membrane, was stable enough for maintaining the integrity of hyperthermophilic pre-cells and for maintaining a definite organism–environment dichotomy over a long period of evolution, perhaps over hundreds of million years. In any case, Wächtershäuser does not seem to consider chirality as an issue [14].

From a chemical point of view, the theory could be experimentally tested on its predictive power, i.e. its power to predict hitherto unknown chemical reactions. Wächtershäuser admits that the reactions demonstrated so far may well be the tip of an iceberg of synthetic reactions for a pioneer organism that has yet to be discovered experimentally. Thus, the main hypothesis of the model, i.e., the possible existence of the so-called “*pioneer organism*” has not yet been confirmed by experimental data. Another issue is, as noticed by Wächtershäuser, that fluid flow through a localized volcanic-hydrothermal flow duct is of a short duration, too short to cover a long period of evolution. To overcome the issue the author hypothesizes that intense ejection and scattering of crustal materials, and their settlement in renewed flow beds would have caused the process to be resumed time and time again with ever more advanced catalytic endowments and cellular organization [14]. JF Allen points out a problem with autocatalysis. The product of a series of reactions is also their substrate. Autocatalysis becomes more plausible if the autocatalytic cycle takes place in a confined space, so that the concentration of its components can build up. Surface catalysis alone cannot easily explain how a reactant becomes concentrated, since the volume of the solute is, for all practical purposes, infinite, being, one supposes, the volume of water in all the oceans of the World. On a flat surface, autocatalysis will tend to be prevented, or quenched, by dilution [14]. Wächtershäuser argues that inorganic compartments exist, and can also be produced, apparently, in the laboratory, as a result of mixing two solutions, which have solutes that react to form a ‘froth’ of insoluble precipitates, citing Russell’s hypothesis that such structures, consisting of vesicles bounded by sulphides of iron and nickel, served as the first ‘incubators’ of life by permitting, containing, and sustaining chemoautotrophic synthesis [15]. Wächtershäuser claims that there is reproduction because some of the synthetic organic products exhibit an autocatalytic positive feedback into the synthetic reactions whence they arise. This is questionable as a true reproduction is not really the multiplication of specific chemical compounds but rather the multiplication of individual systems leading to a population of similar systems. The author adds that evolution occurs because the autocatalytic feedback effect exhibits variations and the existence of an autocatalytic feedback cycle signifies the historic fact of its previous de novo ignition and in this sense, each autocatalytic feedback effect constitutes an instance of inheritance or a memory effect [14]. Such a speech is obscure to me and does not sound very much scientific. Hence, Wächtershäuser’s claim that his model is able to evolve is questionable, at best. According to the author the first stage of cellularization is the formation of acetyl thioester as evolutionary precursor of acetyl-CoA. Then, there would have been a very complex chemical evolutionary path leading to the formation of the first lipids followed by a transition from surface lipophilization to a surface-supported bilayer membrane. Another complex evolutionary path would have followed leading to semi-cellular structures, which would have constituted the beginning of individuation. Finally, again another complex evolutionary path would have led to a full cellularization [14]. This is a nice story, but more a fairy tale than a scientific reasoning.

### Non-templated systems of replicators model

Some authors notice that there are two camps in the origin of life. The metabolism- first camp advocates consider improbable that RNA-like self-replicating polymers appeared before natural selection had operated on chemical networks, whereas genetics-first supporters find implausible the idea that molecular networks without genetic control could have undergone Darwinian evolution. These authors think that a solution to the conundrum can be found in general evolutionary principles shared by some chemical and biological systems [16]. The origin of the first hereditary replicators is still an unsolved problem. On its own, that transition is not evolutionary because, without hereditary replicators, no Darwinian evolution is possible. There should be a gray zone where chemistry and evolution had the first overlap [17]. A scenario should be based on the evolutionary dynamics of populations of replicator molecules because it has the advantage of providing a potential solution to two fundamental problems faced by prebiotic systems at the same time: (i) the physiological problem of overcoming the degradation tendency of any complex molecule, like an oligomer or a polymer; and (ii) the evolutionary problem of transmitting the selective advantages of that complex molecule to the offspring [18]. Thus, a scheme is proposed for how Darwinian evolution could have occurred prior to template replication. The authors use an abstract chemistry based on autocatalytic chemical networks with a particular structure and existing in networks of compartments. These simulations did not confirm previous claims that autocatalytic sets of organic polymer molecules could undergo evolution in any interesting sense by themselves. It was discovered that if general conditions are satisfied, the accumulation of adaptations in chemical reaction networks could occur. These conditions are the existence of rare reactions producing viable cores (analogous to a genotype) that sustains a molecular periphery (analogous to a phenotype) [16]. More recently they state that they believe they have shown limited heredity of a few types hence limited evolvability. They also admit that the system size is unknown. They claim that the most exciting form of evolvability is indefinite, open-ended on going evolution, possibly leading to an increase in complexity, at least in certain lineages [19].

The model in Vasas et al.’s paper traditionally belongs to the metabolism-first scenarios granted they show that there are replicators in their system, but replicator in the strict sense only means an entity capable of autocatalytic growth. Thus, this model has been considered as metabolism-first. William Martin and Eugene Koonin noted the abstractness of the model, which far-removed from real chemistry and real metabolism. The authors also overlook the possibility of preexisting inorganic catalysts that are not part of their reaction products and the possibility that things chemical fall into place more along the lines of thermodynamics than along the lines of mathematics. Moreover, the model requires independent viable autocatalytic cores embedded in a large molecular network as units of evolution [16]. Eugene Koonin argued that it is difficult to understand how and where such large complex molecular networks could have emerged on early Earth and how such a putative non-templated system of replicators could have been stable in real time [16]. In addition, the authors specified that their model would only shown limited evolvability while they claim that the most exciting form of evolvability is indefinite, open-ended on going evolution. Besides the authors don’t address the chiral question compatible with their model.

### Autocatalytic cycles in liposomes model

Vasas et al. say, in terms of “real” chemistry they have been inspired by the concept of autocatalytic protein networks but they mention the model by Fernando and Rowe that shows a similar evolutionary mechanism based on random biomolecular rearrangements rather than ligation and cleavage reactions [16]. Fernando and Rowe quote Maynard-Smith, “*Natural selection is an algorithm that operates in populations of entities capable of multiplication, variation and heredity*” [20]. The question they address is “*what is the simplest ‘machine’ capable of implementing the natural selection algorithm, and how likely was this implementation to arise spontaneously?*” Thus, they opt for an individual- or compartment-first hypothesis based on a population of lipid aggregates. They hypothesize that a geophysical process would have produced such lipid aggregates without specifying the process. The lipid aggregates could have undergone division by externally-imposed agitation and the authors assume that specific intra-lipid aggregate chemical reactions are not required for a base rate of lipid aggregate replication. Then a novel species has one probability of being in the lipid phase, so remaining within the lipid aggregate, and another probability of being in the water phase, so being extruded from the lipid aggregate. During division approximately half of the lipophilic constituents of the lipid aggregate are inherited by each daughter cell. The authors make the assumption that a potential autotrophic reaction exists that is capable of utilizing light energy to drive an otherwise non-spontaneous reaction. They claim segregation of the population into lipid aggregates allows harmful avalanches to be isolated to a compartment. Finally the autocatalytic cycles would have evolved in the liposomes because they were necessary for the maintenance of chemical reaction networks that were beneficial at the liposome level. According to the authors, the system should have shown natural selection at both the level of autocatalytic molecules and lipid aggregates [21]. This model would have been able firstly to show pre- Darwinian evolution and latter on Darwinian evolution.

Surely, their model is less abstract than Vasas et al.’s because it is based on lipid aggregates. While there are indeed possible processes that lead to symmetry breaking the authors do not specify how these processes would have operated in their model and have thus solved the chiral question. In addition, they are unable to specify where on early Earth there would have been a geophysical process-producing lipid aggregates with a potential autotrophic reaction capable of utilizing light energy.

### Inorganic membrane in alkaline hydrothermal vent model

In 1989 Russell et al. had already predicted the properties of deep-ocean alkaline hydrothermal vents more than a decade before their discovery [22]. Then, they suggested the prime exergonic reaction underpinning the synthesis of organic molecules would have been the reduction of CO_2_ itself with electrons stemming from H_2_. The H_2_ driving CO_2_ reduction stems in turn from serpentinization. The fixation of CO_2_ required energy which could have been readily supplied by H_2_ itself, augmented by an ambient chemiosmotic gradient acting across the inorganic membrane and/or the catalytic coupling of exergonic redox reactions to those yielding formate/formyl [23]. At the time of early Earth atmospheric CO_2_ concentrations were much higher than today and molecular oxygen was absent giving very different ocean chemistry from today. High CO_2_ made the oceans mildly acidic (pH 5.5–6) compared with pH 8 today, which, in the absence of O_2_, allowed reduced transition metals, most significantly Fe_2_^+^ and Ni_2_^+^, to accumulate in the early oceans. These metals exhaled from volcanic vents (possibly nearby), gave rise to mineral precipitates at alkaline vents such as silicates, clays, carbonates, and sulfides. For example, at a Lost-City-type vent in an early-Earth setting, this chemistry delivers catalytic Fe(Ni)S minerals. [24]. This model would have followed chemical evolution (i.e., pre-Darwinian evolution) [23].

In favor of this model are the first results of recent experiments with a simple electrochemical reactor to simulate conditions in alkaline hydrothermal vents, allowing that abiotic vent chemistry could prefigure the origins of biochemistry. The precipitation of thin-walled, inorganic structures containing nickel-doped mackinawite, a catalytic Fe(Ni)S mineral, under prebiotic ocean conditions showed the generation of low yields of simple organics while synthetic microporous matrices were able to concentrate organics by thermophoresis over several orders of magnitude under continuous open-flow vent conditions. These experiments provide empirical evidence that simple organics can be generated and concentrated under mild alkaline hydrothermal conditions from H_2_ and CO_2_ using Fe(Ni)S catalysts transected by natural proton gradients in microporous matrices [25]. It is questionable whether these processes could lead to something else than simple inactivated organics. Moreover, there are severe problems for the natural pH gradient hypothesis. Firstly, there is the difficulty of interposing a suitable, stable, thin inorganic membrane between the alkaline vent fluid and the acidic ocean that could hold a sharp pH gradient. At Lost City, there is little or no evidence for the existence of stable inorganic membranes, let alone of membranes holding sharp pH gradients. The second and even more difficult problem is that it is hard to accept that a molecular machine capable of abstracting useful energy from the natural ∆ pH was assembled within the inorganic membrane by chance on the prebiotic earth, that is before the advent of gene encoded proteins and the great power of natural selection. Thirdly, a simple, small, ∆ pH-consuming molecular machine, even if by chance adopting an as yet unknown chemical structure, and a set of barely suspected, facilitating, mechanistic principles, will be unable to function in an inorganic membrane of a thickness deemed acceptable by Russell et al. [26]. Even when the authors supposed that inorganic molecular machines might assemble by chance in the precipitate membranes, and be capable of using the Δ pH to drive unfavourable reduction of CO_2_ by H_2_ to formate and formaldehyde (and indeed, these workers detected both of these compounds in their origin-of-life reaction vessel and contended that was proof of principle for their hypothesis) JB Jackson demonstrated by a straightforward calculation that the formate produced was only that reached on approach to equilibrium without any driving force from Δ pH. Jackson therefore concluded that the reaction was facilitated by isotropic catalysts in the precipitate membrane but not by an anisotropic ΔpH-driven molecular machine [27]. Moreover, the problem of the transition from inorganic to organic membranes is faced. According to Russell et al. the organic takeover from the mineral precursors would have been facilitated by the surface structure of amyloidal peptides between 6 and 10 amino acids long that behave as nests for inorganic clusters. Together these would do the work of proto-enzymes [28]. The authors do not specify how homochiral amino acids would have been produced in their model and about the way their metabolic systems could replicate and transmit their characteristics to the progeny. Therefore the evolutionary path from the mineral precursors to amyloidal peptides is unlikely without solving the chiral issue. Moreover, the evolutionary path from vesicles with membranes composed of amyloidal peptides to the first anaerobic chemolithotrophy organisms using Earth’s inorganic geochemicals is not straightforward without the existence of some kind of Darwinian evolution and in particular of heredity.

### Conclusions on metabolism-first scenarios

The four metabolism-first scenarios discussed above are supposed to show pre-Darwinian evolution. They show major issues as follows: no practical chemical substrate is known (Vasas et al.); experimental data supporting the main hypothesis on which the model is based are lacking (iron-sulfur world, Fernando & Rowe, Vasas et al.) or preliminary (Russell); the source of energy is questionable (Fernando & Rowe’s) or not specified (Vasas et al.); the chiral question is not addressed (Fernando & Rowe, Russell, Vasas et al.) or not considered as an issue (iron-sulfur world); multiplication abilities are not truly present (iron-sulfur world) or unknown (Russell), heredity is not clearly demonstrated (iron-sulfur world) or absent (Russell), a plausible site on early Earth is unknown or not specified (Fernando and Rowe and Vasas et al.); the system is unstable (Vasas et al); the evolutionary path seems either complex (Fernando and Rowe,) or questionable (iron-sulfur world, Russell).

## Replicator-first scenarios: RNA and pre-RNA worlds

The RNA world hypothesis describes an early Earth with self-replicating and catalytic RNA but no DNA or proteins. The possibility of an RNA world was suggested well before Gilbert coined the name in 1986 [29]. Benner et al. have well summarized both the arguments in favor of the hypothesis of an RNA world and the main pitfalls [30]. They noticed that the most direct evidence for an RNA World, crystallographic evidence that the RNA in the ribosome catalyzes peptide bond, is applied within a historical argument, one that infers that all ribosomes in biology use RNA to synthesize peptide bonds, and therefore the last common ancestor of all ribosomes used RNA to synthesize peptide bonds. In the model there would be first pre-Darwinian evolution until the emergence of RNA and then Darwinian evolution.

The idea of an RNA world was actually built upon the diverse roles of RNA in contemporary metabolism and not upon the limited catalytic role of these natural ribozymes. Anyway a major question is how do the first self-replicating ribozymes emerge in the absence of template-directed information replication? [28]. Admittedly, the group of Sutherland shows that activated pyrimidine ribonucleotides can be formed in a short sequence that bypasses free ribose and the nucleobases, and instead proceeds through arabinose amino-oxazoline and anhydronucleoside intermediates [31]. Even among those searching for a prebiotic origin of RNA, Sutherland’s approach is not universally regarded as satisfactory and critics of that type of prebiotic chemistry find that an excessive Deus ex machina, but also note that if amino-oxazole is to be formed in a different locale than glyceraldehyde, the effort to find pH neutral conditions to form amino-oxazole (recognizing the instability of glyceraldehyde at high pH) was unnecessary. If this were not sufficient, the final step requires yet another change of environments, to urea or formamide as a reaction mixture, not water [30]. More recently the same team provided experimental evidence in support of the hypothesis that precursors of ribonucleotides, of amino acids and of lipids have a common chemical origin [32]. In their experiments precursors of ribonucleotides, amino acids and lipids can all be derived by the reductive homologation of hydrogen cyanide and some of its derivatives, and thus that all the cellular subsystems could have arisen simultaneously through common chemistry [33]. In a very recent article Powner et al. demonstrated a divergent, prebiotically plausible reaction strategy for the synthesis of both pyrimidine and purine ribonucleotides on a single oxazoline scaffold [34]. One should nevertheless accept that an important gap remains in our understanding of the processes this could have spontaneously supported the emergence, maintenance and evolutionary potential of such a precellular world. Various chemical reactions traditionally involved in the artificial organic syntheses of RNA precursors are thermodynamically uphill or show high kinetic barriers in the absence of activating molecules or catalysts, respectively [35]. All these experiments use enantiomers in their chemical reactions and thus the chiral question comes back. The origin of life, especially the origin of RNA world, cannot be disclosed without explaining the origin of chiral homogeneity of biomolecules [13]. There is ample research on the chirality of RNA. In particular, J. Garay’s hypothesis is the construction of complexes by the chemo-autotrophic metabolism on mineral surfaces, e.g., the iron-sulfur world proposed by Wächtershäuser [36]. He calls these “*MAR (Metal-Amino-Ribo) complexes*”: a MAR-complex is constituted of “*a metal ion surrounded by co-enzymes (for example, amino acids or short peptides, etc.), and short RNAs*”. His hypothesis does not really lead to a model as such, as the author does not address questions such as: are there any experimental data supporting his hypothesis? What was the energy source? Were there plausible site(s) where the primordial chemical substrates might arise? (Table: “*Strengths and weaknesses of the reviewed scenarios for the origin of life*”). Secondly, the initial chemical substrate of his hypothesis is what he calls a “*MAR-complex*” that seems to answer the chiral question. The author only answers the question if there were MAR-complexes at the very beginning. He likewise answers the questions about replication, heredity, and the evolutionary path from the same hypothesis. The inescapable question is: how could the abiotic emergence of MAR-complexes be explained? Specifically how could the abiotic synthesis of RNA and peptides be explained? While Garay agrees that the origin of the RNA world has not been explained satisfactorily as yet he now supposes that RNA and peptides could have been synthesized abiotically, in particular before the emergence of chirality. From a theoretical perspective, M. Stich et al. say, the current state of knowledge strongly suggests that the emergence of chirality must be based on reactions leading to spontaneous mirror symmetry breaking (SMSB). SMSB are transformations yielding chiral outcomes as non-thermodynamic final stable states, and in the absence of any chiral polarization or external chiral physical forces. This is provided by enantioselective autocatalysis, but not by the simple linear asymmetric induction reactions on which past discussions on deterministic or chance phenomena were based for the justification of biological homochirality. These authors concluded that the racemic outcome results even when the autocatalytic cycles are driven irreversibly by external reagents, in manifestly non-equilibrium conditions. The stability of the thermodynamic limit proves that the racemic outcome is the unique stable state for strictly irreversible externally driven autocatalysis [37]. These theoretical results are clearly in favor of the claim that chiral question is not a trivial question. P. Joshi et al. studied the selective adsorption on the platelets of montmorillonite. They demonstrate that catalytic montmorillonite platelets serve as selective templates for the formation of RNA oligomers. In their experiments the authors start from monomers of nucleotides. Thus, there remains the question of the abiotic synthesis of the monomers [38]. In their experiments for a cross-chiral RNA polymerase J. Sczepanski and G. Joyce began with a population of 10^15^ random-sequence D-RNAs. In their experiments the authors used RNA molecules as ingredients [39]. Hence, again, the question of the abiotic synthesis of RNA is still there. It can be concluded, at least from these publications, that the chiral question is not yet clearly answered. Among insurmountable problems of chemical and informational nature are the unreliability of the synthesis of initial components, the instability of the molecules which increases with chain elongation, the catastrophically low probability of meaningful sequences, the lacking mechanism of the formation of membrane-bound vesicles permeable for the nitrogenous bases and other RNA precursors as well as able to divide on a regular basis [40]. The difficulty in synthesizing nucleotide monomers, not to mention the idea that RNA itself is too complex to have been the first genetic molecule, has focused researchers to search for non-standard molecules as earlier replicators and catalysts and on other means of transitioning to standard nucleic acids. For example, it was proposed the idea that peptide nucleic acids (PNAs) might have preceded RNA nucleotides [41]. This would have been the pre-RNA world. The difficulties remain open in synthesizing the initial components within the paradigm of open far from thermodynamic equilibrium systems and especially because of the chiral question. Thus, it is understandable that Benner et al. note that it is not surprising that Joyce and Orgel called RNA a “*prebiotic chemist’s nightmare*”. Moreover, there is a paradox that all the efforts which have focused on making RNA under a gene first model for the origin of life curiously have come as close as any to providing a working example of a cycle reminiscent of models which put metabolism first. Another limitation of the RNA world has been hypothesized to be the paucity of building blocks in the RNA biopolymer. Certainly, proteins, with 20 amino acids, have a richer diversity of functionality than RNA, and this functionality is useful for binding and catalysis [30]. Finally, as Benner et al. specify it, some have abandoned all genetic biopolymers, suggesting instead that something like Darwinian evolution must have been supported by a set of small organic molecules dissipating free energy in a cycle that through its operation can adapt to changing conditions and evolve in a Darwinian sense [30].

### Conclusions on the RNA and pre-RNA world scenarios

The only replicator-first scenarios discussed above should show pre-Darwinian evolution, actually, firstly pre-Darwinian evolution and secondly Darwinian. There are crucial issues: the source of energy is not specified, the chiral question is not addressed, and a plausible site on early Earth is unknown. In addition, it has its specific issues (see above). An alternative should be therefore found.

## Combined metabolism-replicator scenarios

### Dynamic autocatalytic systems within the dynamic kinetic stability concept (DKS)

Some researchers claim that a chemical environment held in a non-equilibrium state by kinetic barriers constitutes a prerequisite for the development of very specific dynamic chemical systems of constituents and catalysts capable of circumventing these kinetic barriers. They support the concept of the dynamic kinetic stability, DKS [42–44]. According to these authors, the stability kind is the stability associated with entities able to make copies of them at a rate that results in a non-equilibrium steady-state population of replicating entities being maintained over time. The replicated entity, the replicator, is a specific molecule of the autocatalytic metabolic system but not the system per se. The authors claim that when there are several autocatalytic metabolic systems they will tend from less stable (persistent) to more stable (persistent) forms because of a logical law of nature they term the persistence principle, i.e., only the most persistent systems will remain after a certain time. The model would be capable of Darwinian evolution according to the authors.

When a metabolic system is very much persistent it doesn’t mean that its composition is more complex and diversified. It would not be unlikely that simpler metabolic systems were more persistent. The evolution of a given population of metabolic systems toward a population of increasingly complex and diversified systems is not the most likely. According to R. Pascal the solar radiation, with energy maximum in the visible domain precisely corresponding to the requirement for self-organization would be the source of energy for DKS systems [43]. Even if the accumulated evidence suggests that photosynthesis began early in Earth’s history it was probably not one of the earliest metabolisms [45]. The author does not address the chiral question. The authors also found that the replicated entity, the replicator, is a specific molecule of the autocatalytic metabolic system but not the system per se. Hence, it is questionable to claim there is a true reproduction because, as in Wächtershäuser’s model, the system does not multiply. In addition A. Pross and R. Pascal say nothing about heredity [42–44]. Finally, R. Pascal admits that no practical example has yet been identified of an autocatalytic network capable of both reproducing itself, fed by light energy, and carrying information in a way possibly leading to selection and accumulation of genetic information [43].

### Compositional genomes in the lipid world model

At Weizmann in Israel D. Lancet conceptualized a model based on heterogeneous assemblies of diverse lipid-like mutually catalytic amphiphilic molecules [46]. The so-called “*graded autocatalysis replication domain (GARD) model*” utilizes chemical kinetics to simulate the behavior of mutually catalytic sets as an alternative to alphabet-based inheritance. A basic feature in GARD is that non-covalent, micelle-like molecular assemblies are capable of growing homeostatically, i.e. catalytically maintaining the assembly’s composition as it grows (dynamic buffering). The maintained compositional information can be propagated to progeny assemblies upon occasional fission [47]. Energy sources available to drive the synthesis reactions leading to the amphiphilic molecules would range from volcanoes and hydrothermal vents to solar photochemistry and pyrite-dependent reduction [48]. According to the authors their GARD model would have had some capacity for pre-Darwinian evolution [46].

Recently, it has been argued that collectively autocatalytic metabolic networks, such as the GARD, do not allow for fitter compositional genomes to be maintained by selection [49]. The authors have replied, based on a large number of simulations, that when quasi-stationary composomes rather than arbitrary compositions serve as selection targets, GARD networks are capable of a significant response to selection. Importantly, this can happen chiefly when a high proportion of mutual catalysis is present in a GARD network [47]. However, the authors’ hypothesis seems rather unlikely, i.e., that, in the conditions of the prebiotic earth, a high proportion of mutual catalysis could have emerged. In addition, it is questionable that “*compositional information*” could have been transferred to bilayer membrane lipid vesicles when these would have taken over the “*micelle-like molecular assemblies*”. In experiments looking at transitions from micelles to vesicles the fatty acid in micelles are just incorporated into the preformed vesicles without any possibility to transmit a “*compositional information*” present in the micelles [50–52]. Against this argument, according to D. Lancet (see his comment as a reviewer of this article), is that the GARD model would not invoke a capacity of a small micelle to confer its composition onto a much larger vesicular assembly when fusing with it. Rather, it would invoke the possibility that homeostatic growth via single molecule accretion could gradually lead to increasingly larger assemblies having a similar composition to that of the original micelle. Regarding the chiral question the authors described an extension of their Graded Autocatalysis Replication Domain (GARD) model. The new model called Chiral-GARD (or C-GARD) is introduced to explain the symmetry breaking between left- or right-handed biomolecules in the modern biosphere. Their main conclusion is that a strong symmetry breaking may result from a relatively modest asymmetry in mutual (auto)catalytic activity: that is D-enantiomers which are more likely to catalyze other D-enantiomers than L-enantiomers, while L-enantiomers preferentially catalyze their L-brothers and sisters. The C-GARD model would highlight the possibility that chiral selection is a result of, rather than a prerequisite for early life-like processes and thus would not have been an issue [53]. Actually, the authors assume that all molecules in the C-GARD simulation are asymmetric as they claim that for sufficiently complex molecular structures it is justified to assume that essentially all molecules are chiral. Thus, they disregard non-chiral isomers in their simulations. The authors’ assertion seems unrealistic, i.e., that, in the prebiotic environment, there would have been sufficiently complex molecular structures allowing to assume that all molecules were chiral.

### Lipid vesicles in alkaline hydrothermal field model

In accordance with the hypothesis that something like Darwinian evolution must have been supported by a set of small organic molecules dissipating free energy in a cycle that through its operation can adapt to changing conditions and evolve in a Darwinian sense [31] a model based on lipid vesicles with a bilayer membrane was imagined. Considering the general importance and the various roles of compartments in contemporary cells (both in terms of general cell identity and processes) it is very likely that the formation of some type of compartment already occurred in early pre-Darwinian times. Furthermore, most popular are vesicular structures, which currently attract considerable attention as primordial cell compartment models [32]. Such vesicles with a membrane composed of potentially abiotic lipidic amphiphiles could have been produced in alkaline hydrothermal fields akin to a Lost-City-type vent in an early-Earth setting [54]. Their formation could have occurred from abiogenic production of short-chain hydrocarbons [55]. As provided for in abiotic chemical reactions which typically produce very heterogeneous mixtures of compounds the vesicle bilayer membranes would have been composed of mixtures of amphiphiles in such a way that the vesicle membranes become molecularly, compositionally and organizationally highly complex, similarly to the lipid matrix of biological membranes [56, 57]. When hypothesizing a bi-layer membrane composed of a mixture of various distinct amphiphilic compounds there is the opportunity to generate by chance a huge number of theoretically possible combinations of the amphiphiles leading to specific arrangements. Among the combinations, a specific local arrangement of amphiphiles could have appeared in the inner part of the membrane able to catalyze the formation of larger products from small carbon-based molecules. Small molecules could easily penetrate the membrane while larger products would have tended to be retained because of difficulty of the larger produced molecules crossing back out of the membrane. Among larger products there were specific compounds able to catalyze the transformation of the local membrane arrangement into a stabilized membrane site by the formation of covalent bonds (instead of intermolecular H-bonds) most likely between the hydrophilic poles of adjacent amphiphiles. Although the reaction of stabilization was theoretically reversible, practically it was not. This was because there was a very low likelihood of another interaction of the intra-vesicular soluble chemical catalyst with the membrane site due to the dilution of the former in the inner volume of the vesicle. Hence, a hypercycle according to the terminology by Manfred Eigen [58] would have been formed. It was actually a positive feedback produced by mutually catalytic soluble catalyst/membrane site pairs. In the meantime the increasing chemical catalyst concentration in the vesicle would have allowed the formation of new membrane sites by its interaction with the same kind of local membrane arrangements and the sites would have been more or less randomly distributed over the inner surface of the vesicle membrane. When the vesicle divided into daughter vesicles [59] the chemical catalyst and the membrane sites or only one element of the soluble catalyst/membrane site pair was likely to be present in the daughter vesicles leading to the transmission of the hypercycle [10]. Chirality would have occurred by chance when compounds with a chiral centre carbon atome were synthesized from specific small carbon-based molecules. In addition, the model would have had the potential to favor the polymerization of carbon-based molecules, for example leading to the synthesis of peptides [9]. As a reminder, the kind of catalysis in the model mediated solely by small organic molecules is comparable to organocatalysis. Organocatalysis or “*small-molecule catalysis*” is a field recently rediscovered in chemistry that in the past decade has become a thriving area of general concepts and widely applicable asymmetric reactions [60]. C. Barbas believed that this chemistry not only provides for fascinating and efficient syntheses of chiral molecules but also may serve to explain the emergence of homochirality in the prebiotic world [61]. This is one of our main hypotheses already developed in a previous paper [9]. When the chemical catalyst is chiral it is possible to say that once a single chirality is developed, a proto-organism could grow this way, and divide, so propagating the chirality [62]. In the model the polymerization process of the carbon-based molecules (e.g., amino-acids) needs the migration of the sites in the membrane to allow the formation of bi-sites, tri-sites etc. and thus polymerization [9]. When vesicles divide a few bi-sites, tri-sites etc. may be transmitted to the daughter vesicles but cannot be replicated as such. Migration of the sites in the daughter vesicles is needed again to form other bi-sites, tri-sites etc. Hence, this particular point should be tested in a research programme [10]. Darwinian evolution has been implemented in the model since the beginning. The only pre-Darwinian processes at work were the formation of lipid vesicles with multiplication abilities and the selection of the most viable. Before the emergence of hypercycles genetically and ecologically distinct lineages could not yet appear on which natural election might operate and the evolution over time of the composition and complexity of the vesicles was not yet possible. The main issue of the model is the absence of experimental data today supporting the hypothesis that mutual catalysis is possible in lipid vesicles with heterogeneous membranes in the environment of alkaline hydrothermal fields. A research programme has been proposed to confirm the plausibility of the model [10].

### Conclusions on combined metabolism-replicator scenarios

Combined metabolism-replicator scenarios show major issues too: no practical chemical substrate is known (DKS), experimental data supporting the main hypothesis on which the model is based are lacking (all scenarios); the source of energy is questionable (DKS), the chiral question is not addressed (DKS) or considered as not essential (Lancet and Segré), replication is disputed (Lancet and Segré), a plausible site on early Earth is not clearly specified (DKS), the evolutionary path is questionable (DKS) or unlikely (Lancet and Segré) or would have been handicapped in case of a tedious polymerization (Tessera).

## Discussion

The definition of the concept of “*life*” should be discussed when considering the question of prebiotic evolution from relatively simple chemistry to life. The first major difficulty encountered when facing the problem of how life originated is that there is not a general consensus on what life is and on how it should be defined. By contrast, it is clear, since the pioneering work by CR. Woese and GE. Fox, that all the current biodiversity is the outcome of Darwinian evolution from a primitive cellular species, the so-called last universal common ancestor or LUCA [63]. Is it possible to define life scientifically, with strictly chemical and biological criteria? [64]. The same authors find that although finding consensus about the nature and definition of life is a very difficult issue, and will remain as a subject of debate probably for a long time, there is nowadays relatively widespread agreement on which features should be shared by the simplest “*living systems*”. They must possess a genetic apparatus able to store and transmit information to their progeny, some sort of metabolism for gathering nutrients and energy from the environment, and a selectively permeable boundary that separates and distinguishes them from this environment. They deduce that it is necessary to develop chemistries which enable the synthesis of information-bearing polymers, protometabolic networks, and protocellular compartments under compatible prebiotic conditions to explain how the first organisms might have appeared on Earth, or elsewhere. If there is a consensus that the first “*living systems*” should have possessed a genetic apparatus able to store and transmit information to their progeny, i.e., systems with hereditary property, the issue is how protometabolic networks without heredity would have had the capability to evolve towards systems with heredity while Darwinian evolution had not started operating yet [27]. Reviewed scenarios were divided between metabolism-first, replicator-first and combined metabolic-replicator. All scenarios show major issues either because of the lack in the model of any practical example, experimental data, plausible sources of energy, or a convincing explanation of the emergence of chirality. There are indeed possible processes to lead to symmetry breaking. For example, counted among the most noteworthy findings of the last decade in asymmetric catalysis research must certainly be Soai and coworkers’ discovery of asymmetric amplification in the autocatalytic alkylation of pyrimidyl aldehydes with dialkylzincs [65]. In his exciting article Donna Blackmond confirmed the Soai reaction. Amplification of enantiomeric excess (ee) can only result if a means exists to suppress the catalytic action of the “wrong” hand of the catalyst. Experimental studies of the Soai reaction reveal that statistical formation of dimer catalyst species coupled with lower activity of the heterochiral dimer is sufficient to rationalize the evolution of high ee from a tiny initial imbalance. This general mechanism could be effective in a world of simple organic molecules such as those likely to have been present in the prebiotic world. Blackmond might agree that the Soai system represents a “*triumph of reductionism*” in helping us to understand the chemical origin of life in molecular terms [66]. In particular, Blackmond and her team notably highlight that the Soai reaction has inspired a wealth of studies – experimental, theoretical, and computational – aimed at understanding the processes of symmetry breaking and asymmetric amplification. They nevertheless notice that this most prominent experimental example of asymmetric autocatalysis exhibits significant constraints on substrates and reaction conditions. Finally, the search for further examples, in particular for reactions exhibiting chemistry of greater prebiotic relevance, continues apace but has proved inconclusive to date [67]. There are problems too regarding the evolutionary path, information on the mechanism of replication (either inaccuracy or absence of replication), heredity, stability of the systems, existence of plausible sites on early Earth favoring the model emergence, reliability of the synthesis of primordial ingredients, instability of the molecules which increases with chain elongation, catastrophically low probability of meaningful sequences etc.

Regarding the metabolism-first scenarios there are specific issues well emphasized by Abel and Trevors [64]:

- Only physicodynamic bias reduces through self-ordering tendencies the vast sequence spaces needed for prebiotic molecular evolution. Even if vast sequence spaces had been available in a theoretical primordial soup, no known mechanism exists for the prebiotic selection of prescriptive sequences;
- In prebiotic molecular-evolution environment, no differential survival or differential reproduction exists yet. Natural selection in the context of Darwinian evolution does not exist yet in a prebiotic environment;
- The wheel would have to be reinvented with each new generation. Homeostatic metabolism is statistically prohibitive enough as it is as a one-time event;
- No metabolism-first model can be sustained without rapid incorporation of any minimal successes into a recorded, integrated, heritable, cybernetic scheme.

Moreover, a theoretical argument plays against the plausibility of metabolism-first scenarios. The term “*evolution*” is a generic term that can be understood as the change over time of dynamical systems. Thermodynamics drive change (i.e., evolution) in any physical systems, including the diversity of life on earth. There are actually different levels of evolution, from simple to more complex, taking into account the level of interaction between systems and populations of systems (i.e., agents) and their local environments. It is possible to describe four fundamental levels of evolution with the most basic level corresponding to the monotonic degradation of isolated systems, which excludes any interaction with the external environment. When systems interact with the local environment through inputs and outputs they are able to oppose degradation through self-organizing dynamics. Self-organization may be identified as the second level of evolutionary dynamics, because the complexity of self-organization confers much greater persistence on the system. Self-organizing systems tend to grow as they receive environmental inputs, as long as the inputs outweigh the outputs, but growth ultimately destabilizes systems as internal dynamics become uncoordinated. This results in large systems splitting into several smaller systems. Multiplication of self-organizing systems may be identified as the third level of evolution because it leads to populations of systems, which further extends persistence of the system’s dynamical cascade. Only the fourth fundamental level, i.e., Darwinian evolution, is able to promote the emergence of genetically and ecologically distinct lineages on which natural selection may operate [68]. Metabolism-first systems may reach the third level of evolution at best but not the fourth because heredity is lacking. Systems reaching the third level of evolution (i.e., able to multiply themselves) may improve their lifetime but still fully depend on their environment for the evolution over time of their composition and complexity. Without a mechanism of a phenotypic inheritance, which is independent from variation in the local environment, metabolism-first systems cannot sustain any newly acquired composition or complexity. Szathmáry and co-workers notice that stable hereditary propagation is not possible without effective autocatalysis in contrast to reflexively autocatalytic networks, complemented by rare uncatalyzed reactions and compartmentation. The latter networks, resting on the creation and breakage of chemical bonds, can generate novel (‘*mutant*’) autocatalytic loops from a given set of environmentally available compounds. Real chemical reactions, which make or break covalent bonds, rather than mere incorporation of components, are necessary for open-ended evolvability. They conclude that the most exciting form of evolvability is indefinite, open-ended on going evolution, possibly leading to an increase in complexity, at least in certain lineages. They think the following conditions are required: (1) a rich chemical combinatorics, (2) digital inheritance based on template replication, (3) an environment made more complex by evolution itself, and (4) the fact that we cannot pre-state in general the possible preadaptations. How all this could have emerged in early evolution is a nut hard to crack [28]. In the lipid vesicles in alkaline hydrothermal field model the only pre-Darwinian processes at work were the formation of lipid vesicles with multiplication abilities and the selection of the most viable. Without the emergence of genetically and ecologically diverse lineages on which natural election might operate the evolution over time of the composition and complexity of the vesicles was not possible. Only after Darwinian evolution would have emerged by chance in one step with the occurrence of specific arrangements of the amphiphiles among a huge number of combinations in the inner part of the bilayer membrane and thus leading to the emergence of hypercycles. The emergence of Darwinian evolution was impossible as long as multiplication and heritability were lacking. Only Darwinian evolution could have led to an evolution in complexity over time.

The replicator-first scenario RNA/Pre-RNA world seems to solve the previous issues, as multiplication and heritability are present in its more advanced state. However, it encounters its own insurmountable difficulties and remains a “*prebiotic chemist’s nightmare*” even if some of these difficulties seem to have been solved [30, 31, 33, 34]. In addition, all the efforts which have focused on making RNA under a gene first model for the origin of life curiously have come as close as any to providing a working example of a cycle reminiscent of models which put metabolism first. If the RNA and Pre-RNA world were rather metabolism-first models at their very beginning they would have encountered the same problems. As a combined metabolism-replicator scenario the DKS model would have been able to evolve through Darwinian evolution since the beginning according to the authors. The replicator was a specific molecule of the autocatalytic metabolic system but not the system per se. It is questionable to claim there was a true reproduction, as the system did not multiply. In addition Pross and Pascal say nothing about heredity. The two other combined metabolism-replicator scenarios have also their own problems (see above and Table 1). The last scenario has the fewest issues. It only required small carbon-based molecules, i.e., without needing complex molecules like proteins and/or nucleotides at the beginning. It would also have taken the opportunity of a combinatorial of all the possible local molecular arrangements of a heterogeneous lipid membrane composed of a mixture of amphiphiles. Such a combinatorial of the possible local molecular arrangements would have allowed the emergence of a hypercycle based on mutual catalysis and been a source of variation. The lipid vesicles of the model are open far-from-equilibrium systems generated by a continuously supplied flow of matter and energy from the alkaline hydrothermal fields, may multiply, and have hereditary properties. The model would have allowed Darwinian evolution to start operating from the beginning. Its catalyzing properties would have solved the issue of chirality. However, its evolutionary path of the systems would have been handicapped if the polymerization was tedious. The main issue of the model is the present lack of experiments supporting the hypothesis that a hypercycle by mutual catalysis might emerge in lipid vesicles with a heterogeneous membrane in the conditions of a relatively high temperature (90 °C) and high pressures in alkaline hydrothermal vents in salty seawater. This is why a research program has been recently proposed to implement the different steps to support this new direction of research in the field.

In their scenario the authors appeal to either pre-Darwinian or Darwinian evolution to support an evolution over time. Pre-Darwinian scenarios do not seem the most plausible. They may reach the third level of evolution (i.e., able to multiply themselves in addition to involving self-organizing dynamics) but not the fourth level that requires the emergence of genetically and ecologically distinct lineages on which selection may operate. Among the scenarios that appeals to Darwinian evolution from the beginning, one seems to show the best profile although it has its own shortcomings.

## Conclusion

Pre-Darwinian evolution is here defined as an evolutionary continuity between chemistry and Darwinian evolution. It is meant to describe the initial chemical steps before the emergence of Darwinian evolution. Some of the better-known open far-from-equilibrium system-based scenarios are reviewed. No metabolism-first scenario is convincing enough to make such a pre-Darwinian evolution is plausible. Alternatives to metabolism-first scenarios are either replicator-first or combined metabolism-replicator scenarios. The only so-called replicator-first scenarios, the RNA and the Pre-RNA world scenarios, remain a “*prebiotic chemist’s nightmare*”. Other approaches are combined metabolism-replicator scenarios implementing either pre-Darwinian evolution or Darwinian evolution. They nevertheless have their own issues. Among the reviewed models, one shows the least, although it has shortcomings. In particular, its main hypothesis needs to be validated by experimental data. Darwinian evolution is at work from the very beginning in this scenario. From this critical review it is inferred that the concept of “*pre-Darwinian evolution*” appears questionable, in particular because it is unlikely if not impossible that any evolution in complexity over time may work without multiplication and heritability allowing the emergence of genetically and ecologically distinct lineages on which natural selection may operate. Only Darwinian evolution could have led to such an evolution. Thus, Pre-Darwinian evolution is not plausible according to the author. Surely, the answer to the question posed in the title is a prerequisite to the understanding of the origin of Darwinian evolution.

## Reviewers’ comments

### Review #1: P. Lopez-Garcia, Centre National de la Recherche Scientifique, France

The manuscript has some originality and comments on important aspects on the transition from chemistry to biology. I find the style a bit complex and not very clear even if the ideas are interesting (though not necessarily novel). It deserves publication, the literary style could be improved.

*I thank the reviewer for her comments, particularly when she finds that the manuscript has some originality. Regarding the literary style I tried to improve it.*

In this manuscript, M. Tessera critically examines various models on the origin of life and more specifically interrogates about whether these models involve pre-Darwinian (chemical) evolution prior to the Darwinian evolution characteristic of open far-from-equilibrium systems that are considered alive. The subset of chosen models are classed in three categories: metabolism-first, replicator-first and coupled metabolism-replicator models. Many of the criticisms and concerns highlighted by Tessera have been already raised by previous authors; he revisits them in the context of a transition from chemical to Darwinian (biological) evolution. Overall, the ideas summarized in this critical review are stimulating for research on the chemistry-biology transition. I have, however, some comments: - My major comment is that definitions of Darwinian and pre-Darwinian evolution are somewhat fuzzy and this affects whether a system is considered to evolve by Darwinian or pre-Darwinian evolution. The definition of Darwinian evolution is more obvious, this corresponds to encoded (genetically inherited) variation upon which natural selection acts. Pre-Darwinian evolution involves chemical evolution. But does a system where genotype and phenotype are not (yet) coupled (i.e., containing a replicator plus not-encoded components) evolve via pre-Darwinian or Darwinian evolution? For instance, Tessera claims that lipid vesicles produced in the surroundings of alkaline vents and acting as chemical reactors that eventually include replicators display Darwinian evolution since the beginning. However, these initial lipid-vesicle reactors are not encoded (even if there are replicators inside), so in principle, there should equally represent a pre-Darwinian to Darwinian evolution transition as in other models. - I personally find amphiphile-vesicle models involving co-evolving metabolism and replicator systems as the most practically plausible for the origin of life on Earth. However, even if these systems would show Darwinian evolution from the beginning (which I questioned above), does this mere fact (Darwinian evolution from the beginning) qualify them as more likely than models where a pre-Darwinian/Darwinian evolution transition is required? If so, why?

*As I specify it in the “Background” of the manuscript I prefer to use the expression “pre-Darwinian evolution” instead of “prebiotic evolution” because the concept of life is very much debatable according to me, eventually questionable* [8–12] *while the mechanism of Darwinian evolution can be well defined. Thus I choose not to use words like “alive”, “life”, “living organisms”, “biotic”, “prebiotic”* etc. *except when the researchers I cite use them. I would have preferred to use the term “level-4 evolution” instead of “Darwinian evolution” in accordance with the claim that there are four fundamental levels of evolution* [67]*. Unfortunately, the scientific community to refer to it does not yet accept this view. I find questionable the “pre-Darwinian evolution” concept. Consequently, I cannot find an accurate definition of it. According to me, “Pre-Darwinian evolution” may only be defined in reference to the definition of “Darwinian evolution”. I agree with the reviewer that genotype and phenotype should be coupled. For instance, genotype is represented by the membrane sites in my model and phenotype by the carbon-based molecules catalyzed by the latter as they may impact the structure and the functions of the membrane. Genotype and phenotype are clearly coupled as they form a hypercycle. When vesicles multiplied the daughter vesicles inherited both genotype (*i.e. *membrane sites) and phenotype (*i.e.*, carbon-based molecules) either directly when the hypercycle was transmitted to the daughter vesicles or indirectly when only one element was transmitted but able to reconstruct the hypercycle* [10]*. Thus, separate lineages formed on which natural selection might have operated. Once lipid vesicles with multiplication abilities formed Darwinian evolution would have emerged in one step with the occurrence of specific arrangements of the amphiphiles among a huge number of combinations in the inner part of the bilayer membrane. The only Pre-Darwinian processes at work were the formation of lipid vesicles with multiplication abilities and the selection of the most viable. Finally I think only Darwinian evolution could have led to an evolution in complexity over time. To avoid any ambiguity I now clearly answer the question of the plausibility of pre-Darwinian evolution. Of course, I confirm in the manuscript that the answer to the question posed in the title is a prerequisite to the understanding of the origin of Darwinian evolution.*

Why not including Wächtershäuser’s iron-sulfur world in the comparison? I understand that it has severe problems, notably in the transition to cellularization. Nonetheless, this is one of the most influential models and not less problematic than the Russell’s chemical garden. At least, some of its chemical predictions were proved. – Chirality.

*Wächtershäuser’s iron-sulfur world model is now analyzed in the manuscript.*

The fact that many models do not address the issue of chirality does not imply that they may not accommodate an explanation for chirality. The absence of proof is not the proof of absence. I guess that in many of these models, chirality is simply seen as some kind of consequence of chance. Once you start incorporating one particular isomer, the choice was selectively maintained. - This brings me to my last general comment. The role of chance is ignored in this review. Within the realm of biology, Darwinian evolution is not a full synonym of biological evolution because in addition to selective processes, there is genetic drift. What about pre-genetic drift? This is not trivial because even if one gets experimental evidence for a particular model, this does not mean that historically life originated that way. This would only provide an argumental basis not to discard a particular model.

*For sure chirality has emerged luckily. In my model chance would have been at work when mutual catalysis emerged for the first time in lipid vesicles with heterogeneous membranes. Even if the emergence of a mutual catalysis was allowed by the structure of the vesicle membrane composed of a mixture of amphiphiles chance would have played its part. This occurred when a specific arrangement of amphiphiles appeared among the huge number of possible combinations of arrangements. It was able to catalyze the synthesis of a specific carbon-based molecule. This latter soluble compound had the property of catalyzing the transformation of the local membrane arrangement into a stabilized membrane site. Chance would have operated again when specific small carbon-based molecules led to the synthesis of a bigger molecule with a chiral centre carbon atome. Surely, in the other models chance may have played its part too to make chirality appear but the researchers should present a rational and plausible explanation. I agree that genetic drift should have played its part. There is no particular reason why it would not have occurred in my model. I do not understand what the reviewer means by “pre-genetic drift”.*

### Review #2: A. Poole, Stockholm University, Sweden

This manuscript has a promising title. Unfortunately the manuscript seems instead to be more focused on giving a potted critique of the shortcomings of some of the better-known models for the origin of life. The main issues the author highlights are around whether the models adequately address issues like chirality or Darwinian evolution. A more extensive review of the various models would be helpful; as presented, this review part is a bit too uneven, and needs clearer explanations of the proposed models before launching into critique. The section that discusses the question posed in the title is too brief - it is only a few lines on page 13 (lines 1–25), where the author presents four ‘levels’ of evolution. This is not referenced, but it does note that ‘evolution’ is broader than ‘Darwinian evolution’. However, the question posed in the title is not really discussed in any great depth, and, for me, did not expand the existing discussion around this interesting question. The impression I got from reading the article was that the author’s answer to the question he posed was, ‘yes, but it’s not important’.

*Thank to the reviewer I realized that my view on the plausibility of pre-Darwinian evolution was not so clear as there was a misunderstanding in the reviewer’s impression of my opinion about it. This essay highlights critical aspects of the plausibility of pre-Darwinian evolution. It is based on a critical review of some better-known open, far-from-equilibrium system-based scenarios supposed to explain processes that took place before Darwinian evolution had emerged and that resulted in the origin of the first systems capable of Darwinian evolution. Each model was evaluated according to the researchers’ answers to eight crucial questions that should be addressed (*Table 1*). I tried to summarize the models as well as possible by using the researchers’ wording as far as possible but, surely, my reports cannot be fully exhaustive and unbiased. I appreciate the reviewer’s citation of our proposition, G. Hoelzer and I, that there are four fundamental levels of evolution* [67]*. In accordance with our claim I would have preferred to use the term “level-4 evolution” instead of “Darwinian evolution”. Unfortunately, the scientific community to refer to it does not yet accept this view. I find the concept of “pre-Darwinian evolution” questionable. It is unlikely if not impossible that any evolution in complexity over time could have worked without multiplication and heritability. Only Darwinian evolution would have led to such an evolution. By the way the only pre-Darwinian processes at work in the model I propose were the formation of lipid vesicles with multiplication abilities and the selection of the most viable. Only after Darwinian evolution would have emerged by chance in one step with the occurrence of specific arrangements of the amphiphiles among a huge number of combinations in the inner part of the bilayer membrane. To avoid any ambiguity I now clearly answer the question of the plausibility of pre-Darwinian evolution. Of course, I confirm in the manuscript that the answer to the question posed in the title is a prerequisite to the understanding of the origin of Darwinian evolution.*

### Review #3: D. Lancet, Weizmann Institute of Science, Israel

As instructed, I am checking whether the original referee comments have been addressed to satisfactory standards. My own comments refer to the revised version (R1). Reviewer 1 Points 1,3, are satisfactorily addressed by the author. Point 2 (beginning “In this manuscript, M. Tessera critically examines various models on the origin of life…”) is valid, and has not been fully addressed. In the abstract, the authors present the following conclusion: “From this critical review it is (inferred) that the concept of ‘pre-Darwinian evolution’ appears questionable, in particular because it is unlikely if not impossible that any evolution in complexity over time may work without multiplication and heritability. Only Darwinian evolution could have led to such an evolution. Thus, Pre-Darwinian evolution is not plausible according to the author”. How then can the attribute “Pre-Darwinian” appear for any model in the last column of the table, a column entitled “initial evolution”? I read the author’s conclusion above as implying that whatever chemical processes that took place prior to the advent of Darwinian evolution cannot be called evolution at all. This is because the author applies the same necessary criteria (multiplication and heritability leading to complexification) to both pre-Darwinian and Darwinian evolution. Thus if the criteria are not fulfilled, we have neither Darwinian nor pre-Darwinian evolution. But then, to avoid the confusion on which both reviewer 1 and I agree, the title of the paper should be “what chemical processes led to Darwinian evolution”. Point 4 beginning with “The fact that many models do not address the issue of chirality does not imply that they may not accommodate an explanation for chirality”. I fully agree with this comment and feel that it has not been adequately addressed. The many models which have the value “not an issue” and “not addressed” in the column entitles “Chirality issue” of the paper’s Table, attest to the idea that homochirality should not serve as a yardstick for judging evolution of any kind. This is, in fact, supported by the author’s inference based on a paper of ours (Ref 52): “The C-GARD model would highlight the possibility that chiral selection is a result of, rather than a prerequisite for early life-like processes and thus would not have been an issue”. I strongly suggest that the an issue criterion for evolution be eliminated altogether. Reviewer 2 I agree with this reviewer’s comment: “A more extensive review of the various models would be helpful; as presented, this review part is a bit too uneven, and needs clearer explanations of the proposed models before launching into critique”. I wish to support this comment by addressing the example of my own model (GARD), pointing to necessary corrections. The author should please re-check that the description of other models might not have been similarly afflicted. Here are the points that need to be corrected in the description of the GARD model: 1) The author’s statement: “The model is also based on the view that non-equilibrium self-organizing systems have dynamic properties that exist in a state close to chaotic behavior allowing the emergence of autocatalytic cycles…” is not correct. In the GARD model (as in other similar models) the emergence of mutually catalytic networks (not “autocatalytic cycles”, which is a restrictive term) is afforded merely by the nature of the molecules, i.e. their capacity to exert catalysis on each other, irrespective of chaotic behavior. 2) The author says: “…mutually catalytic sets as an alternative to alphabet-based inheritance”. The GARD model has a form of alphabet-based inheritance. The crucial difference between GARD and a templating biopolymer model is that the former accumulates and reproduces compositional information (counts of chemical “alphabet” letters), while the latter encompasses sequence information (order of chemical “alphabet” letters). 3) The current text proclaims: “A basic feature in GARD is that non-covalent, micelle-like molecular assemblies capable of growing homeostatically (i.e., buffered enough as to maintain stability) according to the assembly’s constitution store compositional information can be propagated after occasional fission (i.e., assembly splitting)”. This confusing sentence should better read: “A basic feature in GARD is that non-covalent, micelle-like molecular assemblies are capable of growing homeostatically, i.e. catalytically maintaining the assembly’s composition as it grows (dynamic buffering). The maintained compositional information can be propagated to progeny assemblies upon occasional fission”. 4) The author states, based on ref. 47, that “Regarding the evolvability of the system it has recently been demonstrated that replication of compositional information (in GARD) is so inaccurate that fitter compositional genomes cannot be maintained by selection”. This criticism is hotly disputed, as exemplified in one of our papers [PMID: 22662913], and it would be fair to make this statement less unqualified and quote the alternative view. 5) The author says (based on Refs 49–51): “Moreover, there is no reason why ‘compositional information’ should have been transferred to bilayer membrane lipid vesicles when these would have taken over the ‘micelle-like molecular assemblies’.” This statement is based on a misunderstanding, and should be omitted. The GARD model does not invoke a capacity of a small micelle to confer its composition onto a much larger vesicular assembly when fusing with it. Rather, it invokes the possibility that homeostatic growth via single molecule accretion could gradually lead to increasingly larger assemblies having a similar composition to that of the original micelle. Returning to Reviewer 2 comments: A sweeping negativity of this reviewer is manifested in the statement “…the question posed in the title is not really discussed in any great depth, and, for me, did not expand the existing discussion around this interesting question”. This, in my opinion, is overstated. I feel there is value in this review, warranting publication in Biology Direct.

*I thank the reviewer for his helpful comments. Regarding the GARD model I modified the sentence when I agreed with the reviewer that it was incorrect (*e.g.*, the basic feature in GARD). When a question is disputed I still mention it with the arguments,* i.e.*, the criticisms, and the counter-arguments,* i.e.*, the reviewer’s reply (*e.g.*, the question of evolvability and of the transfer of the compositional information). With regards to the chiral question I cannot agree that homochirality should not serve as a yardstick for judging evolution in complexity over time. While it is not addressed in most models it does not mean that the chiral question is not crucial. As the reviewer noticed, I present the results of his simulations tending to support the reviewer’s view that chiral selection is a result of, rather than a prerequisite for early life-like processes and thus would not have been an issue. However, I also observe that the authors’ assertion supporting the relevance of these simulations seems unrealistic,* i.e.*, that, in the prebiotic environment, there would have been sufficiently complex molecular structures allowing them to assume that all molecules were chiral. Finally, I believe that the new title of the manuscript the reviewer proposes does not suit the aim of the manuscript,* i.e.*, to support the view that Pre-Darwinian evolution is not plausible. According to this view, the likely processes that would have taken place and allowed evolution in complexity over time before Darwinian evolution emerged are not satisfactory. In my model, the only pre-Darwinian processes at work are the formation of lipid vesicles with multiplication abilities and the selection of the most viable. These processes are not sufficient to allow an evolution in complexity over time.*

### Review #4: T. Dandekar, Department of Bioinformatics, University of Wuerzburg, Germany

The paper makes a case that purely pre-Darwinian evolution does not exist, looking at different examples of chemical evolution and the different major aspects they cover. This table presents some new comparative results. The conclusion is that even in chemical evolution models there is some hereditary element involved, so according to the author, some Darwinian evolution. 2. I think it is worthwhile to stress this point and the comparison with the chemical models stresses this point appropriately and that justifies a publication 3. What could be added would be some more implications, for instance if any type of evolution always needs some element of heredity, does this (more information is passed to the next generation) enhance then always the speed of evolution? 3b. Does then determining whether there is evolution always boil down to identify some heritable element (or information storage)? 4. Of course, choosing the suitable definition you can always be right as an author, but probably by such a definition of evolution you largely ignore the more general and bigger class of self-organizing processes, right? 4b. So my main worry is that by defining terms and things as you do, you may get rid of pre-Darwinian evolution (as you claim that there is always heredity necessary), however, you then become blind for the large, interesting and important class of self organizing phenomena in physics and chemistry who happen without any gene storage or any other such direct storage.

*I agree with the reviewer that it would be less constraining for the purposes of the search of the origin of life if pre-Darwinian evolution was plausible. It would open the search to include self organizing phenomena in physics and chemistry which happen without any gene storage or any other such direct storage. I don’t think it is a question of definition, like, for example the definition of life. Darwinian evolution is a mechanism. It works as it allows evolution in complexity over time, It is based, amongst other things, on natural selection on distinct lineages. Without information storage and the possibility of transfer to the progeny, no lineage may emerge. Thus, natural selection cannot operate and allow evolution in complexity over time.*

## Abbreviations

DKS: Dynamic kinetic stability
DNA: Deoxyribonucleic acid
D-RNA: Dextro-ribonucleic acid
ee: Enantiomeric excess
GARD: Graded autocatalysis replication domain
LUCA: Last universal common ancestor
MAR-complex: Metal-Amino-Ribo complexes
RNA: Ribonucleic acid
SMSB: Spontaneous mirror symmetry breaking
